# Estimation of Transfer Function Coefficients for Second-Order Systems via Metaheuristic Algorithms

**DOI:** 10.3390/s21134529

**Published:** 2021-07-01

**Authors:** Omar Rodríguez-Abreo, Juvenal Rodríguez-Reséndiz, Francisco Antonio Castillo Velásquez, Alondra Anahi Ortiz Verdin, Juan Manuel Garcia-Guendulain, Mariano Garduño-Aparicio

**Affiliations:** 1 Industrial Technologies Division, Universidad Politecnica de Queretaro, El Marques 76240, Mexico; francisco.castillo@upq.mx (F.A.C.V.); alondra.ortiz@upq.mx (A.A.O.V.); manuel.garcia@upq.edu.mx (J.M.G.-G.); 2 Red de Investigación OAC Optimización, Automatización y Control, El Marques 76240, Mexico; juvenal@uaq.edu.mx (J.R.-R.); mariano.garduno@uaq.mx (M.G.-A.); 3 Engineering Faculty, Universidad Autónoma de Querétaro, Santiago de Querétaro 76010, Mexico; 4 Information Technology Division, Universidad Politecnica de Queretaro, El Marques 76240, Mexico

**Keywords:** parameter estimation, metaheuristic, Gray Wolf Optimizer, Jaya algorithm, transfer function

## Abstract

The present research develops the parametric estimation of a second-order transfer function in its standard form, employing metaheuristic algorithms. For the estimation, the step response with a known amplitude is used. The main contribution of this research is a general method for obtaining a second-order transfer function for any order stable systems via metaheuristic algorithms. Additionally, the Final Value Theorem is used as a restriction to improve the velocity search. The tests show three advantages in using the method proposed in this work concerning similar research and the exact estimation method. The first advantage is that using the Final Value Theorem accelerates the convergence of the metaheuristic algorithms, reducing the error by up to 10 times in the first iterations. The second advantage is that, unlike the analytical method, it is unnecessary to estimate the type of damping that the system has. Finally, the proposed method is adapted to systems of different orders, managing to calculate second-order transfer functions equivalent to higher and lower orders. Response signals to the step of systems of an electrical, mechanical and electromechanical nature were used. In addition, tests were carried out with simulated signals and real signals to observe the behavior of the proposed method. In all cases, transfer functions were obtained to estimate the behavior of the system in a precise way before changes in the input. In all tests, it was shown that the use of the Final Value Theorem presents advantages compared to the use of algorithms without restrictions. Finally, it was revealed that the Gray Wolf Algorithm has a better performance for parametric estimation compared to the Jaya algorithm with an error up to 50% lower.

## 1. Introduction

Transfer functions are widely used in engineering and other fields to represent physical systems of various natures. With the transfer function, the stability and the control of the system are analyzed. In these works [1,2,3,4,5], a transfer function is used to solve and describe real-life physical phenomena. Identification is essential for industrial processes, as mentioned in [6,7,8,9]. Another example is [10], where a fractional transfer function is used for modeling and control applications, implementing a low-order algorithm for their application.

The previously mentioned works demonstrate the application and versatility of the transfer function; thermal, hydraulic, electric, or hybrid systems can be modeled with these types of functions. The case of second-order transfer functions is unique since they represent a large number of physical systems. For example, investigations [11,12,13,14,15] work with systems described by a second-order transfer function.

The structure of the transfer function is known. However, the function parameters are typically unknown and come from the parameters of the original differential equations model of the system. The estimation of parameters can be carried out through various techniques. An option that presents greater simplicity and ease of implementation is the so-called metaheuristic algorithms [16]. This type of algorithm has the advantage of its relative simplicity and the disadvantage that the process is iterative [16]. The disadvantage does not pose a significant problem in parametric estimation when the process does not require constant updating and its parameters vary very slowly with time.

This work presents the estimation of the coefficients of second-order transfer functions from their response to the step using two metaheuristic algorithms—the Gray Wolf Optimizer (GWO) [17] and the Jaya algorithm [18]. These algorithms were chosen for their speed and because they do not contain parameters of a specific search. Only the search range, the original population, and the number of iterations should be adjusted.

The contribution of the proposed research is the use of the Final Value Theorem (FVT) to accelerate the search of the parameters, improving one of the weaknesses of metaheuristic algorithms. The proposed method can be used for all those systems described by this type of transfer function. Therefore, once the parameters have been obtained, multiple analyses and control techniques can be performed for each system.

The proposed method was tested in a mechanical system, an electrical system, and an electromechanical system to demonstrate the multiple fields in which the method can be applied. For tests, the systems are subjected to a known step input, and the output is measured. The simulation tests were obtained using the Matlab–Simulink environment. Similarly, it was tested experimentally with sampled signals. The results exhibit a parametric estimate with a Root Median Square Root of 0.00214 and 2 between the estimated and real signal using the GWO combined with the FVT.

The rest of the article is organized as follows: In Section 2, the related works are addressed. In Section 3, the description and structure of the second-order transfer function are described. Section 4 presents the metaheuristic algorithms used and the proposed modification applied with signals obtained by simulation. Section 5 describes the results obtained with the real signals. Finally, Section 6 presents the conclusions of the research.

## 2. Related Work

The parameters estimation of a transfer function is a wide-interest problem. There are multiple examples of works oriented to this task, such as the one presented in [19], where the transfer function of an electrohydraulic servo is determined based on the amplitude–frequency characteristics.

The estimation has been developed through different techniques. Heuristic, metaheuristic, and exact methods have been implemented for this task. For example, in [20], the parametric estimation of a transfer function is performed through the frequency response of the system using the System Identification Toolbox from Matlab and the Vector Fitting. In [21] the authors use the Newton iterative identification method for estimating the parameters of a second-order dynamic system. This technique is an example of heuristic algorithms. Another heuristic example is in [22,23], where the authors use the hierarchical Newton with least squares for parameter estimation and the damping iterative parameter identification method. The impulse response is another option for parameter estimation instead of a step response, as demonstrated in studies [24,25,26]. On the other hand, some works explore the identification of parameters through the gradient [27,28].

Another example of estimating the parameters of a transfer function is the one presented in [1], where the authors identify the parameters based on Green’s function and successfully test their proposal with an experimental case. The authors of [29] focus on identifying a Flame Transfer Function to find the Wiener–Hopf inversion motif without obtaining biased results for a feedback system. The authors test different identification methods and compare them with each other to achieve this.

Some works for the identification of the parameters of the transfer function based on the frequency response. For example, in [30], the authors identify the parameters of a high-frequency induction machine from its frequency response. Another example is the research in [31], where the authors use an incomplete frequency response function and modal data to detect structural damage. On the other hand, as in the present investigation, some works exploit the response in the time domain, such as the one presented in [32].

The identification of parameters via metaheuristic algorithms is a widely studied topic. It is possible to find multiple works, such as [2,33], that work with different platforms and algorithms for identification. There are a wide variety of bio-inspired metaheuristic algorithms. In general, this type of algorithm can be adapted to multiple problems and can be easily adapted to work in parallel. However, its solutions are usually approximate and depend on the search space. If the solution is not in the search range, the algorithm will find a locally optimal solution.

Genetic algorithms are the most widely used algorithms. Their use in different fields can be seen from optimizing crude oil operations in refineries [34] to motor control [2]. However, their performance depends on multiple specific key parameters, which are not trivial in their estimation. For that reason, works such as [35] are based on only general parameter algorithms (population, stop condition and search range). Another popular option is the particle swarm optimization algorithm, which has multiple variants and applications. However, in its original form, it tends to achieve crazy optimizations quickly, so some authors have proposed substantial improvements to the algorithm [36]. On the other hand, algorithms such as cuckoo search use the levy flight to find their target [37]. The cuckoo algorithm has shown similar results to those of genetic algorithms with fewer parameters to configure. Nevertheless, their convergence is slow.

For this work, two algorithms were chosen (GWO and JAYA) and tested in multiple physical applications, and have shown a similar performance to genetic algorithms. In particular, the Jaya algorithm has been chosen for being one of the simplest metaheuristic algorithms without any additional parameters. The case of the GWO algorithm requires an intrinsic parameter—the alpha factor. It allows the comparison of two fast and proven metaheuristic algorithms with different characteristics in their original form.

The parametric estimation is made in an exact way without resorting to the metaheuristic algorithms. However, the system is not applied in a general way, as exhibited in the present article. It highly depends on the type of damping factor, which is often unknown; the simplest case of the stable cases to distinguish is the underdamped ξ<1, where the parameters are estimated with Equation (1):

(1) $$\left.\begin{array}{c} t_{p}=\frac{\pi }{\omega_{d}}\\ M_{p}=e^{\frac{-\xi \pi }{\sqrt{1-\xi^{2}}}}, \end{array}\right\}$$

where $t_{p}$ is the peak time, and $M_{p}$ is the maximum overshoot. For the critically damped case ξ=1, and using the criteria of a settling time within 2% of the final response, Equation (2) is used for the parametric estimation.

(2) $$0.02=\left(1+\omega_{n}t_{s}\right)e^{-\omega_{n}t_{s}}.$$

For the overdamped case (ξ > 1):

(3) $$0.02=\frac{s_{2}}{s_{2}-s_{1}}e^{-s_{1}t_{s}}+\frac{s_{1}}{s_{1}-s_{2}}e^{-s_{2}t_{s}},$$

where $s_{1}$ and $s_{2}$ are the real roots in the transfer function denominator. Finally, for the heavily overdamped (ξ >> 1), the response system is based on a dominant pole, and Equation (2) is used to find the domain pole, posteriorly obtained from the rest of the parameters:

(4) $$0.02=e^{-s_{1}t_{s}}.$$

The exact method provides a helpful answer when there is a single function. However, automating the estimation process can be more complex because the technique requires knowing the type of damping that the system has. While it is simple to distinguish an underdamped system, the other cases can be complicated to differentiate from a time response signal. On the other hand, the exact method requires using numerical methods for three cases. For this reason, the present method becomes relevant when there is no more information, and only the entry and exit of the system are known.

The present investigation is considerably different to the works mentioned above. It uses the response in time, which is more straightforward to measure for most physical systems. Unlike heuristic algorithms, it is simpler to implement and easier to adapt to new systems, only varying the search range. Regarding parametric estimation works with metaheuristic algorithms, no relevant work was found on the estimation of transfer functions until the moment of the review. Finally, regarding the exact method, the present method allows the estimation of the parameters of the transfer function without knowing the damping factor. Although distinguishing one underdamped system from the others is simple, distinguishing between the other three cases is not.

## 3. Background and Typical Uses from a Second-Transfer Function

This section aims to describe the second-order transfer function and some systems that are usually represented by it. In addition, a first-order system and a higher-order system are described, which can be represented by an equivalent second-order function. Thus, the second-order transfer function can represent a wide variety of engineering systems. Typically, it has the form expressed in Equation (5):

(5) $$G\left(s\right)=\frac{D}{As^{2}+Bs+C}.$$

Expression (5) represents a large set of physical systems that do not have zeros. If the equation is reorganized and the dynamic response is taken into account, the same equation is rewritten as follows:

(6) $$G\left(s\right)=K\frac{\omega_{n}^{2}}{s^{2}+2\xi \omega_{n}s+\omega_{n}^{2}},$$

where *K* is the dc gain defined as the ratio of the amplitude response and the steady-state when a stable system is excited with a step input, ξ is the damping factor, and $\omega_{n}$ is the undamped natural frequency. On the other hand, the definition of a transfer function is the output over the input as expressed in Equation (7):

(7) $$G\left(s\right)=\frac{Output(s)}{Input(s)}.$$

Therefore, the output of a system is defined as in Equation (8):

(8) $$Output\left(s\right)=\frac{D}{As^{2}+Bs+C}*Input\left(s\right).$$

Considering the above, the vector of unknown parameters is [A,B,C,D]. If the input to the system is a step-type signal of magnitude *a*, the transfer function is rewritten as in Equation (9).

(9) $$Output\left(s\right)=\frac{D}{As^{2}+Bs+C}\frac{a}{s}.$$

On the other hand, the FVT allows us to know the final value of a transfer function without knowing the transient stage. Moreover, the theorem applies to stable systems and is described in Equation (10).

(10) $$\lim_{s\to 0}sF\left(s\right)=\lim_{t\to \infty }f\left(t\right).$$

If the FTV is applied to a transfer function, such as Equation (10), then the final value of the transfer function is described by Equation (11).

(11) $$Output\left(s\right)=\frac{D}{C}a.$$

Therefore, the final value for this type of function is determined by the value of the coefficients *D* and *C*. The FVT was used as a restriction that the algorithms must comply with when searching for the coefficients.

### 3.1. Transfer Function of an Electrical System

**Figure 1:**
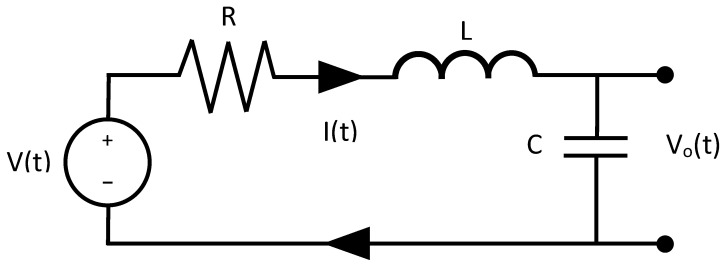
RLC circuit used to obtain the second-order transfer function in electrical systems.

If an RLC circuit, such as the one in Figure 1, is considered, it is known that, using the laws of Ohm and Kirchhoff, the dynamic model of the circuit is determined by Equation (12).

(12) $$V\left(t\right)=V_{o}+LC\frac{d^{2}V_{o}}{dt^{2}}+RC\frac{dV_{o}}{dt},$$

where V(t) is the voltage applied for the source power, I(t) is the current in the mesh, *R* is the resistor value, *L* the inductor value, and *C* the capacitor value.

If the voltage source V(t) is taken as the input and the voltage in the capacitor ($V_{o}$) as the output, then the dynamic model of the circuit is described by Equation (12):

Considering null initial conditions and applying the place transform, the following was obtained:

(13) $$G\left(s\right)=\frac{V_{o}\left(s\right)}{V(s)}=\frac{1}{LCs^{2}s+CR+1}.$$

If a voltage input of magnitude *a* is applied, the expected output of the system is obtained from Equation (14).

(14) $$V_{o}\left(s\right)=\frac{1}{LCs^{2}s+CR+1}\frac{a}{s}.$$

Equation (14) is a second-order transfer function that describes the circuit and, in this case, is known. However, the equivalent expression is obtained directly with metaheuristic algorithms and the standard structure of a second-order transfer function, without the dynamic model or its parameter values. This is particularly useful when the system details are unknown, and only its output can be measured.

**Figure 2:**
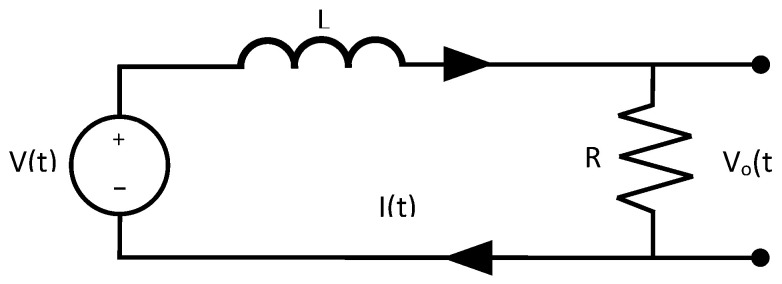
RL circuit used to obtain the first-order transfer function in electrical systems.

Another example in electrical systems is the RL circuit (Figure 2). The RL circuit is modeled as a first-order transfer function. The output of the system is taken as the voltage in the resistor. The differential equation that represents the dynamic model of the RL circuit is expressed in Equation (15), and the corresponding transfer function is exhibited in Equation (16).

(15) $$V\left(t\right)=V_{o}+\frac{R}{L}\frac{dV_{o}}{dt}$$

(16) $$G\left(s\right)=\frac{V_{o}\left(s\right)}{V(s)}=\frac{R}{Ls+R}.$$

### 3.2. Transfer Function of Mechanical Systems

**Figure 3:**
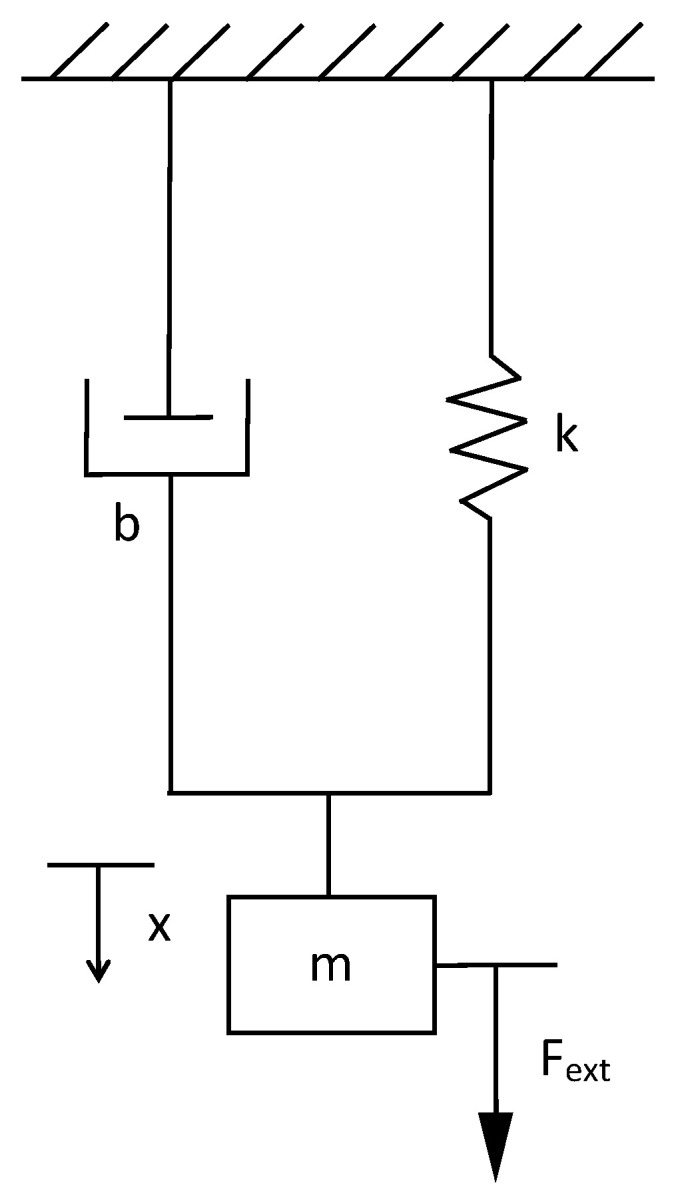
Mass-spring-damper to obtain the second-order transfer function in mechanical systems.

A mass-spring-damper (MSD) system is used to exemplify the transfer function in a mechanical system (Figure 3). The dynamic model is obtained using the second law of Newton, leading to the model described in Equation (17).

(17) $$m\frac{d^{2}x}{dt^{2}}+b\frac{dx}{dt}+kx=F_{ext},$$

where $F_{ext}$ is the external force, *x* is the displacement, *m* is the mass, *b* is the coefficient of friction, and *k* is the spring constant. The transfer function of the system is expressed by Equation (18).

(18) $$G\left(s\right)=\frac{X(s)}{F_{ext}\left(s\right)}=\frac{1}{ms^{2}+bs+k}.$$

**Figure 4:**
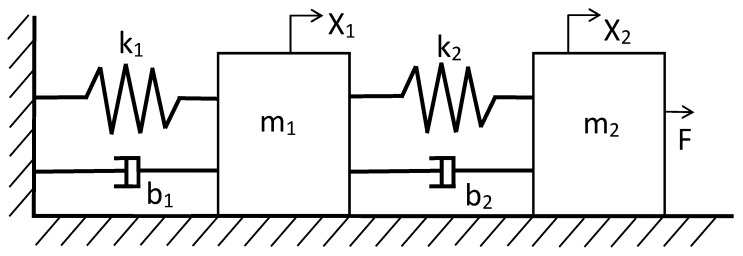
Mass-spring-damper to obtain the high-order transfer function in mechanical systems.

In the mechanical system, the application of the proposed algorithm becomes especially useful since the measurements of the variables and the coefficients tend to be more complex than in an electrical system. The coefficient of friction and the spring constant are often particularly difficult to obtain and require specific tests. Another mechanical system is that displayed in Figure 4. This type of system is a mechanical high-order (ho) system; the differential equation that describes the mechanical circuit is Equation (19) and its transfer function for the displacement in $m_{1}$ is expressed in Equation (20).

(19) $$\left.\begin{array}{c} m_{1}\ddot{x_{1}}=k_{2}\left(x_{2}-x_{1}\right)+b_{2}\left(\dot{x_{2}}-\dot{x_{1}}\right)-k_{1}x_{1}-b_{1}\dot{x_{1}}\\ m_{2}\ddot{x_{2}}=F-k_{2}\left(x_{2}-x_{1}\right)-b_{2}\left(\dot{x_{2}}-\dot{x_{1}}\right), \end{array}\right\}$$

(20) $$G\left(s\right)=\frac{X_{1}\left(s\right)}{F(s)}=\frac{b_{2}S+k_{2}}{l},$$

where *l* is $m_{1}m_{2}s^{4}+\left(b_{2}m_{1}+m_{2}\left(b_{1}+b_{2}\right)\right)s^{3}+\left(m_{2}\left(k_{1}+k_{2}\right)+k_{2}m_{1}-b_{2}^{2}+b_{2}\left(b_{1}+b_{2}\right)\right)s^{2}+\left(k_{2}\left(b_{1}+b_{2}\right)-2b_{2}k_{2}+b_{2}\left(k_{1}+k_{2}\right)\right)s+k_{2}\left(k_{1}+k_{2}\right)-k_{2}^{2}$. Although the system contains a zero, its order is still greater than 2.

### 3.3. Transfer Function of an Electromechanical System

**Figure 5:**
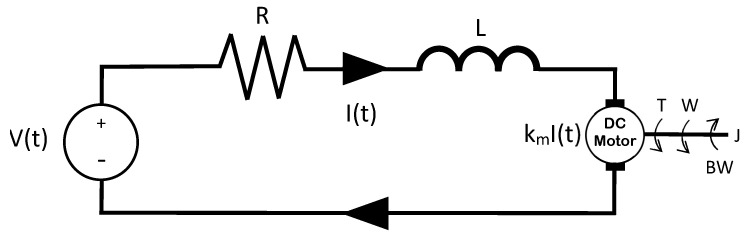
DC motor scheme used to obtain the second-order transfer function in electromechanical systems.

A common electromechanical system is the direct current motor composed of an electrical component and a mechanical component, as shown in Figure 5. Its model is made up of two differential equations, one expression is the electrical part, and the other is the mechanical part, as exhibited in Equation (21).

(21) $$\left.\begin{array}{c} V\left(t\right)=RI\left(t\right)+L\frac{dI(t)}{dt}+K_{e}\omega \left(t\right)\\ k_{m}I\left(t\right)=J\frac{d\omega (t)}{dt}+B\omega \left(t\right), \end{array}\right\}$$

where V(t) is the voltage, I(t) is the current, *R* is the armature resistance, *L* is the armature inductance, $K_{e}$ and $K_{m}$ are the constant electrical and mechanical, respectively, *J* is the momentum of inertia, and *B* is the friction coefficient.

If both motor equations are combined, and with zero initial conditions, it is possible to obtain the transfer function that relates the speed to the voltage, as exhibited in Equation (22). This function is one of the most used for control since it allows the control of a mechanical variable, such as the speed of an electrical input such as voltage.

(22) $$G\left(s\right)=\frac{\omega (s)}{V(s)}=\frac{K_{m}}{LJs^{2}+\left(RJ+LB\right)s+\left(RB+K_{m}K_{a}\right)}.$$

The direct measurement of some of these parameters is complicated or invasive. Consequently, parametric estimation techniques are used.

## 4. Parameter Estimation with Simulated Signals via Metaheuristic Algorithms

In Section 3, it is observed that each system has a transfer function with different coefficients but with the same structure. In contrast, the models expressed with differential equations are based on distinct principles. In the case of hybrid models, a model of differential equations is used to obtain the model. However, it is possible to represent all these models with a second-order transfer function.

**Figure 6:**
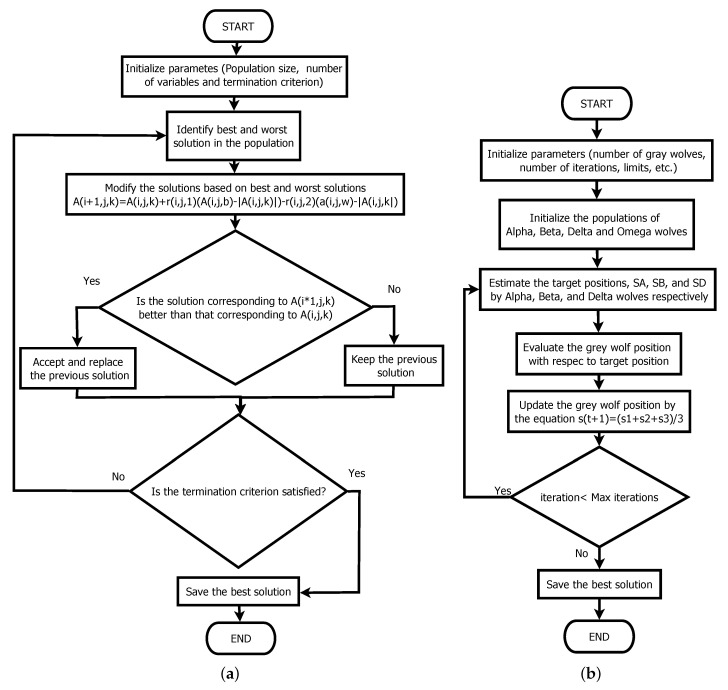
Metaheuristic algorithms used: (**a**) Gray Wolf Optimizer; (**b**) Jaya algorithm.

The Gray Wolf Optimizer [17] and the Jaya algorithm [18] were decided on for the parametric estimation, because both algorithms have high convergence speed and results that are similar to those of genetic algorithms. They also have the advantage of not requiring the adjustment of any specific search parameter, both algorithms having fully equivalent general parameters. The comparison of both algorithms is displayed in Figure 6.

The standard procedure was carried out for the estimation. A step type input was used to excite the system and analyze its response. Therefore, the output of the system and its input are known. Later, the parametric estimation was carried out by employing metaheuristic algorithms.

The cost function for all cases is the Root-Mean-Square Error (RMSE) between the estimated signal and the measured signal. The parameter cost function calculates the similarity between the estimated output signal and the real output signal. Hence, the RMSE represents a measure of error throughout the entire signal. All simulations were run with 100 iterations and 50 individuals for the two algorithms. The parameters are described in Table 1.

**Figure 7:**
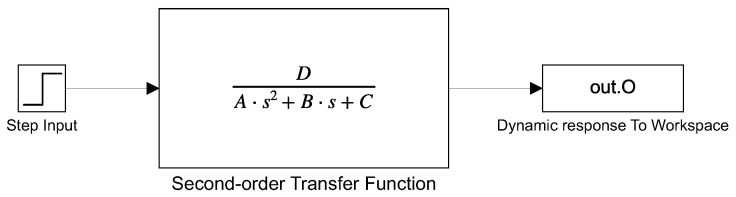
Simulation for obtaining the step response of a second-order transfer function for all systems.

The simulation was carried out using the Matlab–Simulink environment. Simulink simulates the transfer function and Matlab executes the metaheuristic algorithms. The performance of each algorithm was measured to determine which has the best performance. An instability detection system was added to Simulink, based on the magnitude of the signal and the oscillations it presents, to rule out solutions that tend to instability. All Simulink simulations were performed with the simulation displayed in Figure 7, which uses the general structure of a second-order transfer function.

**Figure 8:**
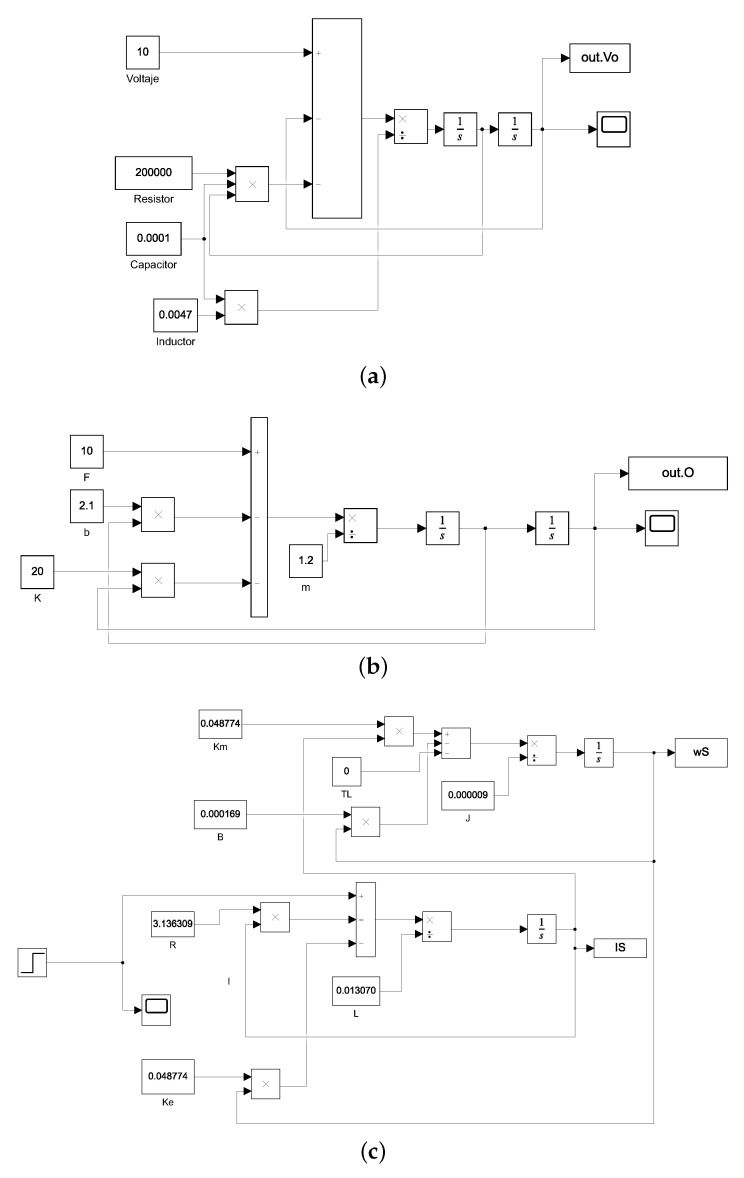
Simulations used for generating the step response signal. (**a**) Simulation for step response of RLC circuit; (**b**) Simulation for step response of mass-spring-damper system; (**c**) Simulation for step response of electromechanical system.

Different simulations were run to obtain the step time response. The values to simulate each system are summarized in the Table 2. The output was obtained by programming the dynamic model in Simulink as exhibited in Figure 8. The simulations were used in both algorithms (Jaya and GWO) to obtain the output of the second-order transfer function’s time response.The numerical method, ODE 45, was used for the simulations with a minimum step of $1\times 10^{-10}$.

**Figure 9:**
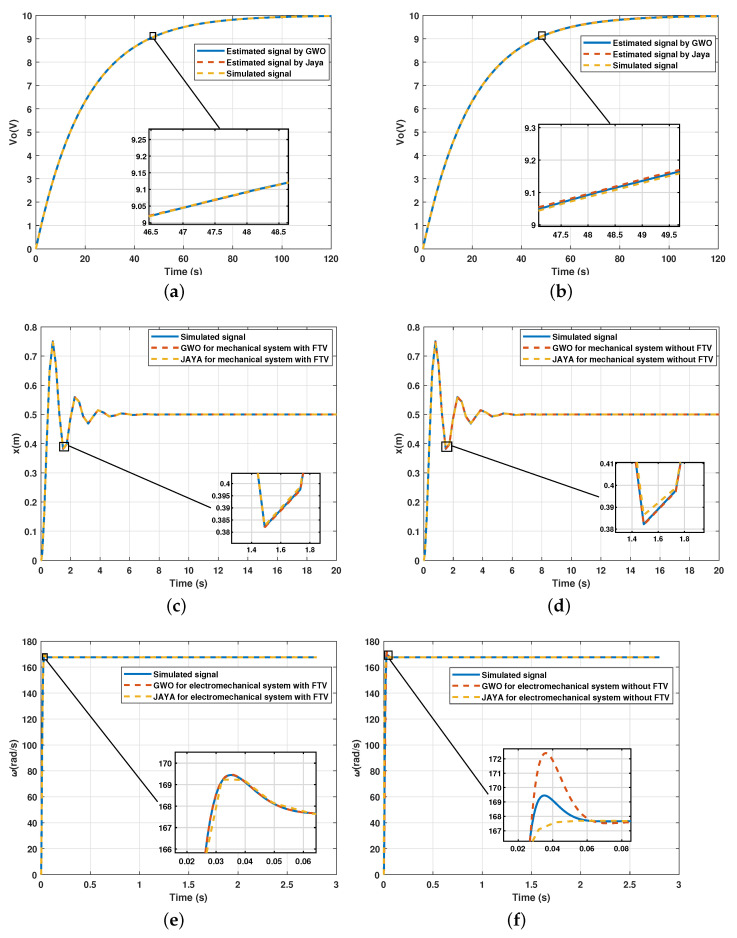
Comparison between estimated signal and simulated signal in second-order systems: (**a**) Algorithms using the FVT as a constraint for the RLC system; (**b**) Unconstrained algorithms for the RLC system; (**c**) Algorithms using the FVT as a constraint for the MSD system; (**d**) Unconstrained algorithms for the MSD system; (**e**) Algorithms using the FVT as a constraint for the DC motor system; (**f**) Unconstrained algorithms for the DC motor system.

The parameter estimation results for second-order systems and the comparison with the use of the FTV are shown graphically in Figure 9 and numerically in Table 3.

**Figure 10:**
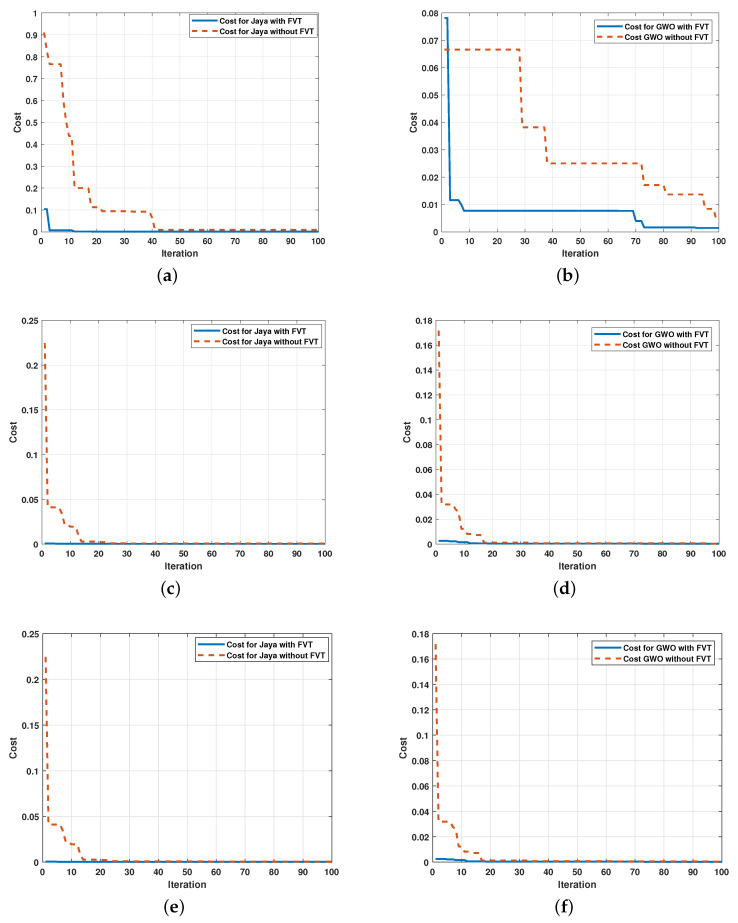
Convergence speed of metaheuristic algorithms: (**a**) Comparison of performance of the Jaya using the FTV in the RLC system; (**b**) Comparison of performance of the GWO using the FTV in the RLC system. (**c**) Comparison of performance of the Jaya using the FTV in the mechanical system; (**d**) Comparison of performance of the GWO using the FTV in the mechanical system; (**e**) Comparison of performance of the Jaya using the FTV in the electromechanical system; (**f**) Comparison of performance of the GWO using the FTV in the electromechanical system.

Although signals that are graphically very similar are observed, a more significant difference is noted if the values of the RMSE in each parameter are analyzed (see Table 3). The convergence speed of each algorithm is displayed in Figure 10 and show the effect of using the FVT.

**Figure 11:**
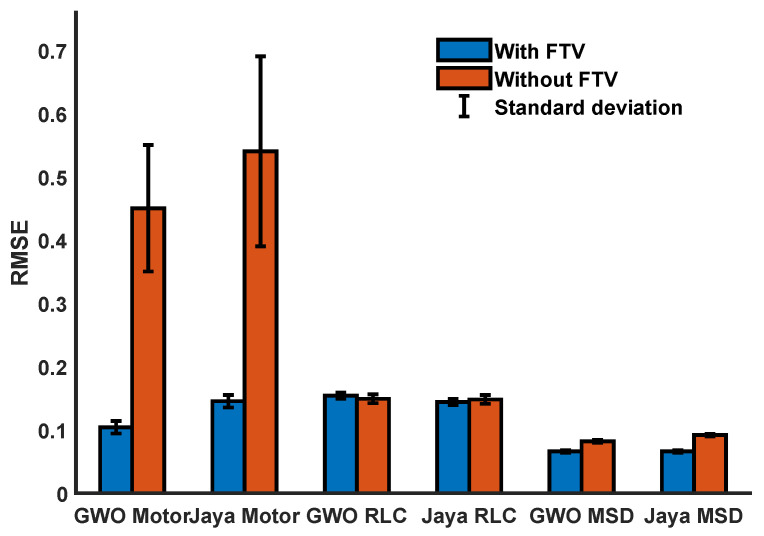
Comparison of the average values of the RMSE and the standard deviation for a set of ten simulations for each type of system.

The positive effect of using FTV is observed in the decrease of the RMSE in the first iterations. Due to the random nature of the metaheuristic algorithms, it is necessary to validate the performance of the proposed method statistically. Therefore, 120 simulations were executed, half of them without FTV, to verify the performance in the second-order systems. The RMSE average and standard deviation are shown in Figure 11.

**Figure 12:**
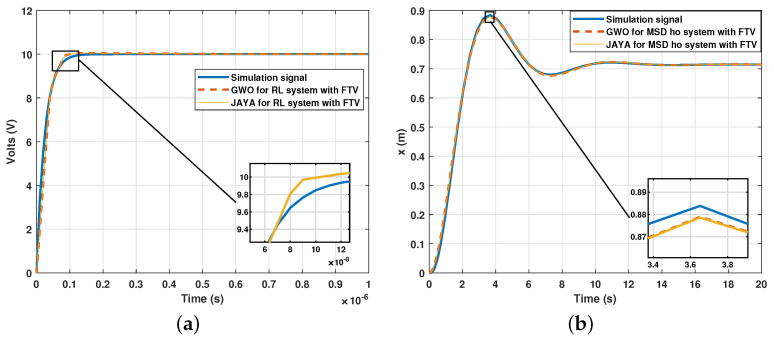
Result for different order systems via metaheuristic algorithms using FTV: (**a**) First order system (RL circuit); (**b**) High-order system (MSD ho).

On the other hand, and to verify the adaptability to the method, a first-order (RL circuit) and a high-order (MSD ho) system was probed with the metaheuristic algorithms and the FVT. The results of the tests are shown in Figure 12.

The response of the RL system is extremely fast when removing the capacitor. It allows the evaluation of the proposed method in systems with very different response speeds. The RMSE between both signals was 0.2283 for the Jaya algorithm and 0.2284 for the GWO algorithm. On the other hand, the RMSE for the MSD ho system was 0.0064 for the Jaya and 0.0063 for the GWO.

The above results indicate that the system can be adapted despite being a lower order function or a high-order system and can calculate an equivalent second-order transfer function. The proposed method has been successfully tested in systems of different natures and orders, in addition to systems with varying levels of damping. The Jaya algorithm tends to 0 in denominator coefficients. Therefore, the cost was artificially modified when the three coefficients in the denominators tend to have a value of zero.

## 5. Parameter Estimation with Real Signals

**Figure 13:**
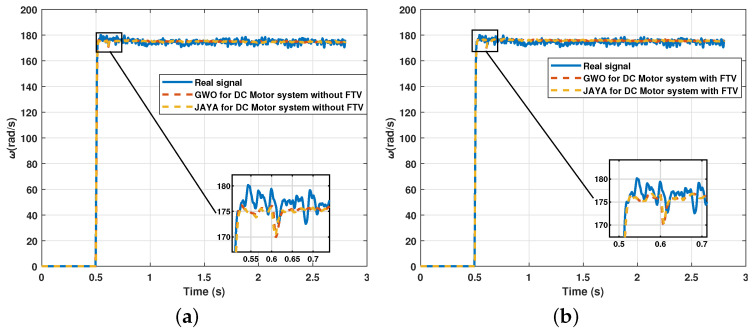
Comparison between estimated signal and real measured signal in the electromechanical system: (**a**) Comparison of Jaya and GWO & FTV for the electromechanical system with real signals; (**b**) Comparison of Jaya and GWO for the electromechanical system with real signals.

**Figure 14:**
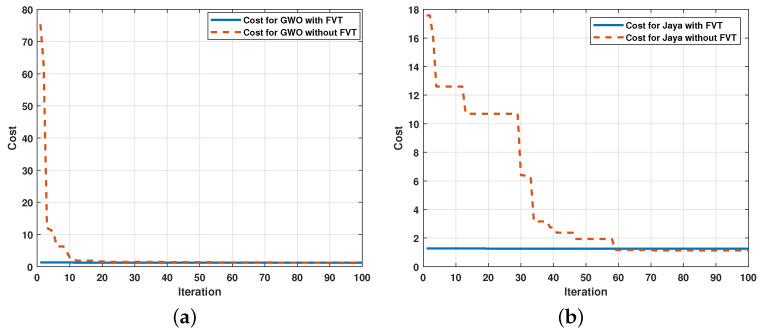
Convergence speed of metaheuristic algorithms for the real electromechanical system: (**a**) Comparison of the performance of the Jaya using the FTV in the real electromechanical system; (**b**) Comparison of the performance of the GWO without the FTV in the real electromechanical system.

In this section, the parameter estimation tests are carried out starting from signals obtained experimentally from the electromechanical system analyzed in the previous section. The acquisition of real data implies noise added by the precision of the hardware used. The Mavilor motor was used with the known nominal parameters described in Table 2 to observe the performance of the metaheuristic algorithms against this type of signal. The signal was acquired with an ADC with a sampling period of 0.001 s, employing the integrated quadrature encoder. Knowing the parameters allows evaluation of the performance of the algorithms for the parametric estimation of a function of second-order transfer in a real test. The results are shown in Figure 13 and the convergence speed is displayed in Figure 14.

**Figure 15:**
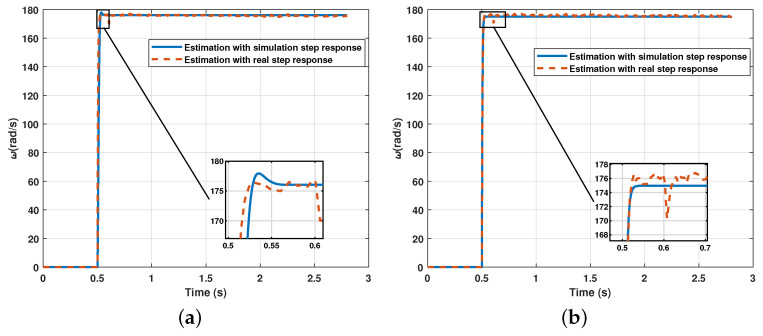
Comparison of the result between the estimation of the transfer function from a real signal and the estimation from a simulated signal: (**a**) Estimation with GWO and FTV; (**b**) Estimation with Jaya and FTV.

The results show that the algorithm can also recreate the signal of the transfer function with real signals. However, the error that increases its effect is expected since the measurement of the signal itself provides a greater degree of uncertainty. Finally, Figure 15 shows the comparison of the result between the estimation of the transfer function from a real signal and the estimation from a simulated signal.

Again, the Jaya algorithm tends to acquire first-order values, making it lose efficiency compared to the GWO algorithm, which correctly estimates values for the A coefficient. Its worst performance is for first-order functions, although they continue to be acceptable values. The use of FVT was beneficial regardless of the nature of the system since the function structure does not change. The results indicate a high precision in the parametric estimation, showing RMSE errors ranging from 0.0006 to 0.1451 when the FTV is used and an error up to 3.64 when it is not considered. This represents a reduction of up to 25 times the RMSE.

## 6. Conclusions

In this investigation, a parametric estimation of a second-order transfer function via metaheuristic algorithms was developed. The general structure of a transfer function and its adaptation to systems of different natures is presented. The method could represent equivalent functions of first-order systems and higher-order systems. The parametric estimation of the parameters was carried out with the Jaya and GWO algorithms. This research proposed to perform a parameter estimation with a high degree of precision for second-order systems.

Additionally, it was verified that the use of the Final Value Theorem as a restriction in the parameter search with two algorithms accelerated the convergence. All the tests carried out presented an increase in the convergence rate, especially in the first iterations, accelerating the processing speed of these algorithms and improving one of their weakest points, which is the execution time.

The main advantage is that this method represents a general way to predict the behavior of any stable system regardless of its order or type, or its dynamic model. The proposed method will find an equivalent second-order transfer function that can accurately predict the behavior of the system in the face of a change in the input.

The disadvantage of the proposed method is that it does not identify the order of the function or the type of damping from the signal. The proposed method has a clear disadvantage compared to the exact method, where it is possible to identify what kind of damping the open-loop system has. Since we are working with transference functions, all zero initial conditions are considered. Another consideration is also only starting with signals from stable systems, the method being inappropriate for highly oscillating systems or systems that tend to instability.

The two metaheuristic algorithms used both exhibit similar results. It should also be remembered that the algorithms start from random numbers, therefore the results vary slightly from one simulation to another, which means that the minor numerical differences presented in this research should not be extensively considered. Finally, all metaheuristic algorithms have problems with local optimum. The search ranges must be adapted in order to avoid falling into a local optimum for this work, changing these ranges to typical, expected values. Finding a relationship between the search ranges and the dynamic response would be beneficial to the method. The Jaya algorithm has a greater tendency to local minimums close to the lower limit. Thus, the use of GWO is recommended for this type of parametric estimation.

## Figures and Tables

**Table 1:** General parameters for Jaya and GWO algorithms.

System	Population	Iterations	Cost Function	Upper Limit	Lower Limit
RLC circuit				[$1\times 10^{-5}$ 100 10 10]	[0 0.1 0.1 0.1]
RL circuit				[$1\times 10^{-5}$ 1 $4\times 10^{5}$ $4\times 10^{5}$]	[0 0 0.01 0.01]
Mechanical	50	100	RMSE	[2.4 4.2 40 40]	[0 0 0.1 0.1]
Mechanical ho				[200 200 200 200]	[0 0 0 0]
Electro-mechanical				[$1\times 10^{-6}$ $1\times 10^{-3}$ 0.1 0.1]	[0 0 0 0]

**Table 2:** Values used for step simulation in different systems.

System	Parameters	Input	Time
RLC	R = 200 kΩ L = 4.7 mH	10 V	120 s
	C = 100 μF
RL	R = 200 kΩ L = 4.7 mH	10 V	1 μs
Mechanical	m = 1.2 Kg k = 20 $\frac{N}{\mathrm{mm}}$	10 N	20 s
	b = 2.1 $\frac{\mathrm{Ns}}{\mathrm{mm}}$
Mechanical ho	$k_{1}$ = 14 $\frac{N}{\mathrm{mm}}$ $k_{2}$=20.8 $\frac{N}{\mathrm{mm}}$	10 N	20 s
	$m_{1}$ = 5.91 Kg $m_{2}$ = 6.48 Kg
	$b_{1}$ = 12.8 $\frac{\mathrm{Ns}}{\mathrm{mm}}$ $b_{2}$ = 20.6 $\frac{\mathrm{Ns}}{\mathrm{mm}}$
Electromechanical	Ra = 3.136 Ω La = 13.07 mH	10 V	2.8 s
	TL = 0 Nm Km = 0.048774
	Ke = 0.048774 B = 0.169 $\frac{{\mathrm{gm}}^{2}}{s^{2}}$
	J = 9 μNm		

**Table 3:** Numerical results for second-order systems.

	RMSE			
System	GWO	Jaya	GWO & FTV	Jaya & FTV
RLC	0.0147	0.01127	0.0165	0.00214
MSD	0.0006	0.0015	0.0006	0.0008
Electromechanical	0.892	3.64	0.0104	0.1451

## Data Availability

No new data were created or analyzed in this study. Data sharing is not applicable to this article.
